# Relation of LAT1/4F2hc expression with pathological grade, proliferation and angiogenesis in human gliomas

**DOI:** 10.1186/1472-6890-12-4

**Published:** 2012-02-28

**Authors:** Zhen Haining, Nobuyuki Kawai, Keisuke Miyake, Masaki Okada, Shuichi Okubo, Xiang Zhang, Zhou Fei, Takashi Tamiya

**Affiliations:** 1 Department of Neurological Surgery, Faculty of Medicine, Kagawa University, 1750-1 Ikenobe, Miki-cho, Kita-gun, Kagawa, 761-0793, Japan; 2 Department of Neurosurgery, Xijing Hospital, Fourth Military Medical University, 127 Changle West Road, Xi'an, Shaanxi, 710032 China

## Abstract

**Background:**

LAT1/4F2hc heterodimeric complex is a major route for the transport of large neutral essential amino acids through the plasma membrane. Although it has been shown that LAT1/4F2hc is highly expressed in a variety of human tumors including gliomas, and LAT1 over-expression is associated with glioma grade and poor prognosis of glioma patients, the precise tissue location of LAT1/4F2hc in gliomas and the precise role of LAT1/4F2hc in glioma biological features remain unclear.

**Methods:**

In the current study, the expressions of LAT1, 4F2hc, CD34 and Ki-67 were investigated by immunohistochemistry in 62 cases of human brain glioma; LAT1/4F2hc expression level, Ki-67 labeling index (Ki-67 LI) and microvessel density (MVD) were measured semi-quantitatively; and the correlation of LAT1/4F2hc expression with histopathological features, Ki-67 LI and MVD in gliomas was further analyzed.

**Results:**

The results showed that both LAT1 and 4F2hc were expressed in all examined specimens. LAT1 but 4F2hc expression levels significantly correlated with the pathological grade and both expression levels significantly correlated with Ki-67 LI of gliomas. We also demonstrated that both LAT1 and 4F2hc immunoreactivity were observed in tumor cells as well as vascular endothelia; furthermore, the LAT1 expression level was markedly associated with glioma MVD as well.

**Conclusion:**

LAT1/4F2hc over-expression is closely correlates with the malignant phenotype and proliferation of gliomas, and LAT1 was associates with glioma angiogenesis. LAT1/4F2hc, especially LAT1, may become a novel potential molecular target for glioma biological therapy.

## Background

In malignant tumor cells, a steady and sufficient sugar and amino acid supply is necessary to maintain a high degree of energy metabolism and protein synthesis for rapid growth and continuous proliferation, and such a supply is supported by the up-regulation of transporters specialized for those nutrients [1]. In terms of glucose supply, the over-expression of glucose transporter-1 (Glut-1) is related to increased glucose uptake in human tumors [1]. Transporters for essential amino acids are particularly important because they are indispensable for protein synthesis to maintain cell integrity and cell cycle progression. Among several amino acid transporters, system L, a Na^+^-independent amino acid transport system, is a major route for providing cells with large neutral amino acids, including branched or aromatic amino acids, which contain most of the essential amino acids [1,2]. Large (L)-type amino acid transporter 1 (LAT1) is a membrane glycoprotein, subserving system L, and was newly cloned in rat glioma C6 cells [3]. LAT1 is highly expressed in brain capillaries that form the blood brain barrier relative to other tissues [4]. LAT1 requires another cell surface glycoprotein, the 4F2 heavy chain (4F2hc), for its functional expression and forming a heterodimeric complex. LAT1/4F2hc complex, one form of 4F2 antigen or CD98 antigen, preferentially transports large neutral amino acids, such as leucine, isoleucine, valine, phenylalanine, tyrosine, tryptophan, methionine, and histidine, which are essential for cell survival and proliferation [1-3].

An increasing amount of research shows that LAT1/4F2hc is over-expressed in a variety of human tumor cell lines and tumor tissues, suggesting that LAT1/4F2hc is implicated in the growth and proliferation of multiple types of human tumor [1,2,5], including lung cancer [6,7], breast cancer [8], esophageal carcinoma [9], prostate cancer [10], uterine cervical carcinoma [11], ovarian cancer [12], thymic carcinoma [13], oral cancer [14], head and neck squamous cell carcinoma [15], metastatic tumor [16], and so forth. For glioma, the most common primary malignant neoplasm arising in the central nervous system, at present, only a few reports exist regarding the LAT1/4F2hc expression in this kind of tumor [17,18]. The related data indicate that LAT1/4F2hc is highly expressed in human gliomas, and associated with glioma progression as well as the poor prognosis of glioma patients [17]; furthermore, treatment of 2-aminobicyclo-(2,2,1)-heptane-2-carboxylic acid (BCH), a system L selective inhibitor, exerts significant cytotoxic effects, with reduced proliferation and increased apoptosis rates in glioma cells with high LAT1 expression [18]. Nevertheless, the exact tissue location of LAT1/4F2hc in gliomas and the precise role of LAT1/4F2hc in glioma malignant biological features remain unclear. Therefore, we investigated systematically the LAT1/4F2hc expression in 62 cases of human brain glioma by immunohistochemistry, and further analyzed the relation of LAT1/4F2hc expression with the glioma histopathological grade, malignant proliferation and angiogenesis in this study.

## Methods

### Patients and specimens

Sixty-two patients with gliomas treated at the Department of Neurological Surgery of Kagawa University from October 1995 to February 2010 were enrolled in this study. All patients had received neither chemotherapy nor radiotherapy before tumor surgical resection. All specimens were obtained from their initial surgery. Patients included 31 men and 31 women ranging in age from 3 to 89 years (mean age: 51.4 ± 18.0, median: 52). Histological subtypes and pathological grades of tumors were defined according to the World Health Organization (WHO) criteria in 2007, as follows: 4 cases with grade I, all specimens were pilocytic astrocytomas; 14 cases with grade II, including 12 diffuse astrocytomas (2 gemistocytic astrocytomas), 1 ependymoma and 1 oligoastrocytoma; 13 cases with grade III, including 12 anaplastic astrocytomas and 1 anaplastic ependymoma; 31 cases with grade IV, all specimens were glioblastomas. Eighteen cases were low grade gliomas, including grade I and II; 44 cases were high grade gliomas, including grade III and IV. The cellular proliferation activity of the tumor was determined by measuring the Ki-67 labeling index (Ki-67 LI), which was obtained routinely by immunohistochemical staining with anti-Ki-67 at the Department of Pathology of Kagawa University.

### Immunohistochemistry for LAT1, 4F2hc and CD34

Immunohistochemical staining was performed on paraffin sections using the polymer-peroxidase method. Briefly, the 3-μm thick sections were deparaffinized and rehydrated routinely. For LAT1 and CD34 immunostaining, antigen retrieval was performed by boiling the sections in 0.01 M citrate buffer (pH 6.0) in a microwave oven for 10 and 15 minutes, respectively. For 4F2hc immunostaining, there was no need for antigen retrieval. After the sections were rinsed in distilled water, the endogenous peroxidase was inactivated with 3.0% hydrogen peroxide in distilled water for 10 minutes at room temperature. After rinsing the sections in phosphate-buffered saline (PBS, pH 7.4), the nonspecific binding site was blocked with 10% normal goat serum for 20 minutes at room temperature. The blocking serum was discarded and then the primary antibodies were added directly. Rabbit antihuman LAT1 polyclonal antibody (Trans Genic, Kumamoto, Japan) was diluted to 1:12.5, rabbit antihuman 4F2hc polyclonal antibody (Trans Genic) 1:8, and rabbit antihuman CD34 monoclonal antibody (Abcam, Cambridge, UK) 1:150 in PBS, respectively. The sections were incubated for 2 hours at room temperature. After rinsing in PBS, goat antirabbit polymer-peroxidase complex (EnVision+/HRP; DakoCytomation, Glostrup, Denmark) was added, and the sections were incubated for 30 minutes at room temperature. After rinsing with PBS, the peroxidase reaction was performed using 0.02% 3,3'-diaminobenzidine tetrahydrochloride (DAB; Sigma-Aldrich, St. Louis, MO, USA) in PBS containing 0.01% hydrogen peroxide for 3-15 minutes at room temperature. The sections were counterstained with Mayer's hematoxylin, then dehydrated and mounted. The primary antibodies were substituted with PBS as a negative control for each section.

### Evaluation of staining results of LAT1 and 4F2hc

The immunoreactivity of LAT1 and 4F2hc in tumor cells was graded semi-quantitatively from (-) to (+++) on the basis of both the percentage of positive cells and the intensity of staining, almost according to the methods described by Nawashiro et al. [17]. This grading procedure was performed by a blinded observer (HNZ) who had no knowledge of the pathological diagnosis and clinical data in all the tumor specimens. Briefly, in the most strongly stained tumor cell area of each section, more than 1,000 tumor cells were observed in high-powered fields at × 400 magnification. The percentage of LAT1 and 4F2hc positive cells was calculated by dividing the number of positively stained tumor cells by the total number of tumor cells observed. The cases that contained equal to or less than 50% positive cells were called less/equal 50% staining, and those that contained more than 50% positive cells were called over 50% staining. The intensity of staining was recorded as negative, weak or strong respectively. Because of the heterogeneity of staining intensity of the tumor cells, the intensity of staining of each case was determined according to the staining intensity of most cells. The cases that contained a weak less/equal 50% staining or unstained specimens were considered negative (-), weak over 50% staining (+), strong less/equal 50% staining (++), and strong over 50% staining (+++). Immunoreactivity of LAT1 and 4F2hc in tumor vessel endothelia was graded semi-quantitatively from (-) to (+++) as well, on the basis of the number of stained microvessels (NMV). Briefly, in areas of the most intensely stained vascular endothelial cells, individual microvessels were counted on three to five × 200 magnification fields. Any endothelial cell or endothelial cell cluster was considered a single countable microvessel. NMV was expressed as the mean value of the absolute number of microvessels per × 200 field for each case. The cases in which NMV ≤1 were considered no staining microvessel (-), 1 < NMV ≤15 few staining microvessels (+), 15 < NMV ≤30 more staining microvessels (++), and NMV > 30 abundant staining microvessels (+++). The highest immunoreactivity grades of LAT1 and 4F2hc in tumor cells or in tumor vessel endothelia were considered their respective ultimate immunoreactivity grades in each case.

### Evaluation of microvessel density (MVD)

The microvessel density (MVD) of gliomas was measured according to the method described by Zhen et al. [19]. Briefly, in areas of the most intense CD34 positive neovascularization, individual MVDs were made on a × 200 magnification field. Any endothelial cell or endothelial cell cluster was considered a single countable microvessel. MVD was expressed as the absolute number of microvessels per × 200 field for each case.

### Statistical analysis

Data were presented as mean, range, and standard deviation (SD). The expression of LAT1 and 4F2hc scores in relation to tumor pathology was evaluated using *χ*^2^-test. The correlations between expression levels of LAT1 and 4F2hc with clinical data, Ki-67 LI and MVD were analyzed using the non-parametric Spearman's rank correlation. Differences of Ki-67 LI and MVD in different pathological grade groups were compared using one-way analysis of variance (ANOVA). Differences of Ki-67 LI and MVD in benign and malignant glioma groups were compared using Student's *t *test. Probability values of < 0.05 were considered statistically significant. All the statistical analysis was performed using the SPSS 16.0 package.

### Ethics Statement

The use of human brain tumor samples for this study was approved by the Kagawa University, Faculty of Medicine Human Subject Ethical committees and followed the guidelines of the declaration of Helsinki, Informed consent was obtained from all patients. Only samples that were initially taken for diagnostic purpose were secondary used for the present study.

## Results

### Qualitative immunohistochemical analysis for LAT1 and 4F2hc

**Figure 1:**
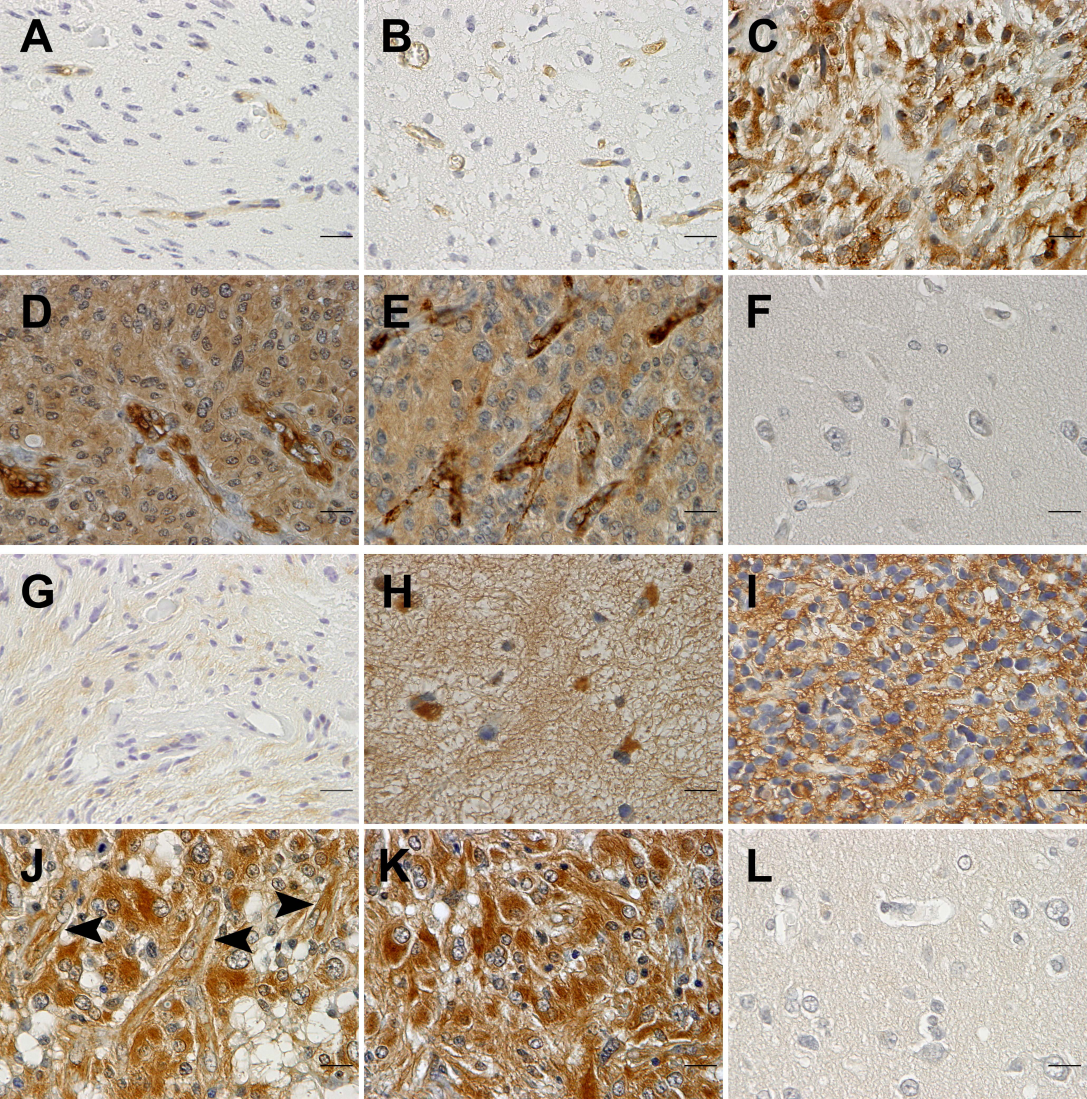
**LAT1 and 4F2hc expression in human brain gliomas and normal brain tissues**. Upper two panels (**A-F**) for LAT1 expression and lower two panels (**G-L**) for 4F2hc expression. (**A**) Pilocytic astrocytoma (WHO grade I) with few LAT1 staining microvessels (graded +). (**B**) Fibrillary astrocytoma (WHO grade II) with more LAT1 staining microvessels (graded ++). (**C**) Anaplastic astrocytoma (WHO grade III) with strong over 50% LAT1 staining in the cytoplasm and plasma membrane of tumor cells (graded +++). (**D**) Glioblastoma (WHO grade IV) with strong over 50% LAT1 staining in cytoplasm and plasma membrane of tumor cells and more LAT1 staining microvessels (graded +++). (**E**) Glioblastoma (WHO grade IV) with strong over 50% LAT1 staining in the cytoplasm and plasma membrane of tumor cells and abundant LAT1 staining microvessels (graded +++). (**F**) Normal brain tissue adjacent to the glioblastoma (**D**) with only negligible LAT1 staining. (**G**) Pilocytic astrocytoma (WHO grade I, the same specimen as shown in A) with weak over 50% F2hc staining in the cytoplasm and plasma membrane of tumor cells (graded +). (**H**) Gemistocytic astrocytoma (WHO grade II) with strong less/equal 50% 4F2hc staining in the cytoplasm and plasma membrane of tumor cells (graded ++). (**I**) Anaplastic astrocytoma (WHO grade III) with strong over 50% 4F2hc staining in the plasma membrane and cytoplasm of tumor cells (graded +++). (**J**) Glioblastoma (WHO grade IV) with strong over 50% 4F2hc staining in the cytoplasm and plasma membrane of tumor cells and more 4F2hc staining microvessels (arrow heads, graded +++). (**K**) Glioblastoma (WHO grade IV, the same specimen as shown in E) with strong over 50% 4F2hc staining in the cytoplasm and plasma membrane of tumor cells (graded +++). (**L**) Normal brain tissue adjacent to the glioblastoma (**E **and **K**) without obvious 4F2hc staining. Immunohistochemical polymer-peroxidase method was used, immunoreactions were visualized with 3,3'-diaminobenzidine tetrahydrochloride (DAB) and nuclear counterstaining was performed with Mayer's hematoxylin. Original magnification × 400, and all bars = 20 μm.

Both LAT1 and 4F2hc were expressed in all the glioma tissues examined (Table 1). LAT1 immunoreactivity was mainly observed in the vascular endothelium and the tumor cytoplasm, as well as the tumor plasma membrane (Figure 1A-E). 4F2hc was predominantly expressed in the tumor cytoplasm and the plasma membrane, as well as in the vascular endothelium (Figure 1G-K). In general, the intensity of 4F2hc immunostaining was higher than that of LAT1 in the tumor tissues. In 11 cases of normal brain tissue adjacent to the tumor, there was only negligible LAT1 and 4F2hc expression, and the immunoreactivity location of LAT1 and 4F2hc in normal brain tissues was similar to that in the tumor tissues (Figure 1F, L). There was a significant positive correlation between the LAT1 expression level and 4F2hc expression level in this series of glioma specimens (*r *= 0.319, *P *= 0.011).

**Table 1:** LAT1/4F2hc expression, Ki-67 LI, and MVD in human brain gliomas

Glioma	LAT1 (no.)			4F2hc (no.)			Ki-67 LI (%)	MVD (no.)
			
	+	++			++	+++		
Grade								
I	1	2	1	1	2	1	1.4 ± 0.8	64.3 ± 15.4
II	6	4	4	3	7	4	3.4 ± 1.9	53.4 ± 54.4
III	5	3	5	2	4	7	13.1 ± 9.4	58.0 ± 29.2
IV	1	4	26	1	7	23	37.2 ± 18.4	77.6 ± 39.9
*P *value*	0.002		0.06					
Degree								
Low	7	6	5	4	9	5	2.9 ± 1.9	55.8 ± 48.2
High	6	7	31	3	11	30	30.1 ± 19.6	71.8 ± 37.8
*P *value*	0.008		0.01					

Total	13	13	36	7	20	35	22.2 ± 20.6	67.2 ± 41.4

Note: LAT1: large (L)-type amino acid transporter, 4F2hc: 4F2 heavy chain, Ki-67 LI: Ki-67 labeling index, MVD: microvessel density

*P value was evluated using *χ*^2^-test

### Correlation of LAT1 and 4F2hc staining with histopathological features

Although the LAT1 immunoreactivity enhanced significantly with the glioma pathological grade ascending (*P *= 0.002), the 4F2hc immunoreactivity did not correlated with the glioma grade (*P *= 0.06) (Table 1). The expression levels of both LAT1 and 4F2hc in high grade glioma tissues were significantly higher than those in low grade glioma tissues (*P *= 0.008 and *P *= 0.01, respectively) (Table 1). However, there was no significant relation between LAT1 and 4F2hc expression with age (*r *= 0.156, *P *= 0.226; *r *= 0.195, *P *= 0.142) and sex (*r *= 0.013, *P *= 0.919; *r *= 0.093, *P *= 0.621) of this series of glioma patients.

### Correlation of LAT1 and 4F2hc staining with Ki-67 LI

**Figure 2:**
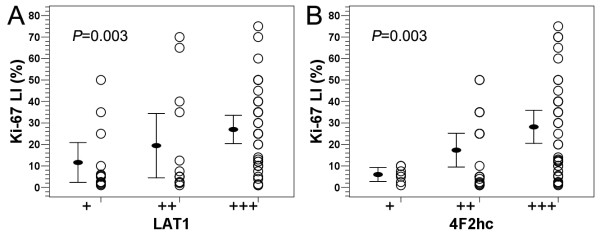
**Correlation of LAT1 and 4F2hc expression with Ki-67 LI in human brain gliomas**. (**A**) A significant positive correlation was observed between LAT1 expression and Ki-67 LI. (**B**) A significant positive correlation was also observed between 4F2hc expression and Ki-67 LI. Spearman's rank correlation test was used.

Ki-67 LI ranged from 1% to 75% with mean 22.2 ± 20.6% in this series of glioma specimens (Table 1). Ki-67 LI increased significantly with the glioma pathological grade ascending (*F *= 25.328, *P *= 0.000), as well as Ki-67 LI in high grade glioma tissues being significantly higher than that in low grade glioma tissues (*t *= 5.842, *P *= 0.000). There was a significant positive correlation between the immunoreactivity of both LAT1 and 4F2hc and Ki-67 LI (*r *= 0.370, *P *= 0.003; *r *= 0.375, *P *= 0.003) (Figure 2).

### Correlation of LAT1 and 4F2hc staining with MVD

**Figure 3:**
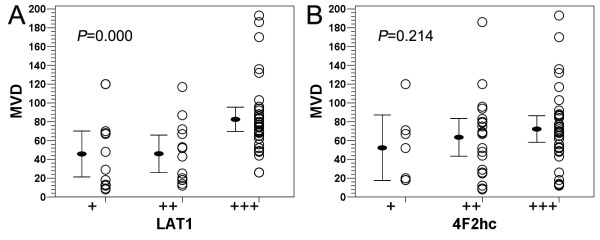
**Correlation of LAT1 and 4F2hc expression with MVD in human brain gliomas**. (**A**) A significant positive correlation was observed between LAT1 expression and MVD. (**B**) No significant correlation was observed between 4F2hc expression and MVD. Spearman's rank correlation test was used.

MVD ranged from 8 to 193 with mean 67.2 ± 41.4 in this series of glioma specimens (Table 1). There was no significant difference in MVD among the different glioma grades (*F *= 1.432, *P *= 0.243). Although MVD in high grade glioma tissues was slightly higher than that in low grade glioma tissues, there was no statistical significance (*t *= 1.398, *P *= 0.167). There was a significant positive correlation between the LAT1 immunoreactivity and MVD (*r *= 0.472, *P *= 0.000), however not for 4F2hc (*r *= 0.160, *P *= 0.214) (Figure 3).

## Discussion

In the present research, we showed that both LAT1 and 4F2hc were over-expressed in human brain gliomas; however, both of them were only negligibly expressed in adjacent normal brain tissues, suggesting that LAT1 and 4F2hc co-expression is closely associated with the formation and development of gliomas. These results were almost in agreement with previous reports [17,18]. Although some previous reports have shown that LAT1 and 4F2hc are co-expressed in brain tissue vascular endothelia of mouse, rat, and cattle [20-22], to date, there is no research showing that LAT1 and 4F2hc are co-expressed in vascular endothelia of human normal tissues or human tumor tissues. To the best of our knowledge, this is the first report demonstrating that both LAT1 and 4F2hc were expressed in vascular endothelia of human brain gliomas, indicating that the LAT1/4F2hc complex plays an important role in large neutral amino acids permeating the blood-brain barrier (BBB) in human brain gliomas. In our previous research with less glioma samples, although the LAT1 immunoreactivity was clear in vascular endothelia in glioma tissues, the 4F2hc immunoreactivity was not obvious [23]. One possible reason was that the 4F2hc immunoreactivity in glioma cells was intensive, so that the 4F2hc immunoreactivity in vascular endothelia was hidden. In this study, although both LAT1 and 4F2hc were expressed in both tumor cells and vascular endothelia of glioma tissues, and there was a significant association between LAT1 expression and 4F2hc expression, the expression level and location of LAT1 and 4F2hc was not completely parallel, i.e. in general, the 4F2hc immunostaining was more intensive than LAT1 immunostaining in glioma tissues, and moreover, LAT1 immunoreactivity was markedly stronger than 4F2hc immunoreactivity in vascular endothelia. On the contrary, 4F2hc immunoreactivity was markedly stronger than LAT1 immunoreactivity in tumor cells, and these results suggest there are different expression regulation mechanisms for LAT1 and 4F2hc [1,2,5].

In the present study, LAT1 but 4F2hc expression levels significantly correlated with the glioma pathological grade, although they were obviously higher in high grade gliomas than in low grade gliomas. Moreover, both LAT1 and 4F2hc expression levels were strongly associated with Ki-67 LI of glioma tissues. These results indicate that LAT1/4F2hc may play an important role in the malignant proliferation and progression of high-grade gliomas [17,18]. The up-regulated expression of LAT1/4F2hc may benefit by providing tumor cells with essential amino acids for high levels of protein synthesis associated with cell activation or hormonal stimulation and also to support rapid growth or excessive proliferation [1,2]. In light of both LAT1 and 4F2hc expression in vascular endothelia, it is reasonable to further analyze whether there is a certain correlation between LAT1/4F2hc expression and angiogenesis. We demonstrated here, also for the first time, that the LAT1 expression level was significantly associated with MVD, although unlike that for 4F2hc expression, in human brain gliomas. These results indicate that LAT1 may play a key role in the neovascularization of gliomas. Therefore, it is involved in multiple aspects for the mechanism of LAT1 promoting the growth and proliferation of glioma cells, that is to say, LAT1 can transport essential amino acids not only from the extracellular fluid to intracellular pool, but also from the tumor microvessel to extracellular matrix [20-22].

In this series of glioma specimens, Ki-67 LI markedly correlated with the glioma pathological grade, and Ki-67 LI in high grade glioma tissues was significantly higher than that in low grade glioma tissues, suggesting that tumor cell proliferative activity obviously enhances with the increase in the glioma pathological grade, which was in agreement with the previous reports [19]. However, there was no statistically significant correlation between the MVD and glioma pathological grade, although MVD in high grade glioma tissues was slightly higher than that in low grade glioma tissues, and if grade I pilocytic astrocytomas are excluded, there was an increasing tendency of MVD with the glioma pathological grade ascending, suggesting that the pilocytic astrocytoma is a very specific glioma subtype with better prognosis, the lowest pathological grade and very low Ki-67 LI, but abundant neovascularization; also indicating Ki-67 LI may more objectively reflect the malignant degree of gliomas compared with MVD. Interestingly, the LAT1 expression level significantly correlated with not only Ki-67 LI but also MVD in this series of glioma specimens, perhaps indicating that the LAT1 expression level can display more biological features of gliomas, and LAT1 may become a novel valuable molecular marker for gliomas.

According to our findings, LAT1/4F2hc was over-expressed in human brain gliomas, and their over-expression was associated with the malignant progression and excessive proliferation of gliomas; furthermore, LAT1 may also play some roles in glioma angiogenesis. Combining the previous related reports that LAT1 over-expression predicts short survival of glioma patients [17], blocking the activity of LAT1 with a system L selective inhibitor can markedly inhibit the growth of glioma cells and induce considerable apoptosis of glioma cells [18], as well as the expression distribution features of LAT1/4F2hc whereby LAT1 is only definitely expressed in few human normal tissues or organs including the placenta, testis and bone marrow, whereas 4F2hc is ubiquitously expressed in various human normal tissues or organs [5]. We consider that LAT1 may become a better potential molecular target for glioma biological therapy compared with 4F2hc.

## Conclusions

LAT1/4F2hc over-expression is correlates with the malignant progression and proliferation of high-grade gliomas, and LAT1 also associates closely with glioma angiogenesis. LAT1/4F2hc, especially LAT1, may become a novel potential molecular target for glioma biological therapy.

## Abbreviations

LAT1: Large (L)-type amino acid transporter 1; 4F2hc: 4F2 heavy chain; Ki-67 LI: Ki-67 labeling index; MVD: Microvessel density.

## Competing interests

The authors declare that they have no competing interests.

## Authors' contributions

HZ, KM, MO, and SO carried out the immunohistochemical assays. HZ participated in the design of the study, performed the analysis and wrote the manuscript. NK conceived of the study, and participated in its design and coordination and helped to draft the manuscript. XZ, ZF, and TT reviewed the manuscript for important intellectual content. All authors read and approved the final manuscript.

## Pre-publication history

The pre-publication history for this paper can be accessed here:

http://www.biomedcentral.com/1472-6890/12/4/prepub
